# β - but not γ-secretase proteolysis of APP causes synaptic and memory deficits in a mouse model of dementia

**DOI:** 10.1186/1750-1326-7-S1-L9

**Published:** 2012-02-07

**Authors:** Robert Tamayev, Shuji Matsuda, Luciano D’Adamio

**Affiliations:** 1 Department of Microbiology & Immunology, Albert Einstein College of Medicine, Bronx, New York, USA; 2 Institute of Cellular Biology and Neurobiology, National Council of Research of Rome, Italy

## Background

Amyloid deposition of Aβ peptide characterizes AD. Aβ derives from sequential cleavage of APP by β- and γ-secretases. Interestingly, mutations in either *APP* or the γ-secretase genes *PSEN1/2* cause familial AD (FAD). Mutation of *BRI2/ITM2b* causes an AD-like familial dementia (FDD) with amyloid deposits. The FDD plaques contain Aβ and ADan, which derives from processing of mutant BRI2 protein by pro-protein convertases. Since amyloidogenic peptides are believed to cause dementias, transgenic mice carrying mutant *APP*, *PSEN1/2* or *BRI2/ITM2b* are used to model these dementias, as over-expression is necessary to reproduce amyloidosis. However, over-expression of mutant genes might produce harmful effects unrelated to dementias and lead to erroneous information concerning pathogenesis and therapy of human diseases. The clinical failures of compounds efficacious in transgenic models support this hypothesis.

## Methods

To avoid artifacts of over-expression, we generated a knock-in mouse model of FDD (FDD_KI_) that, like FDD patients, is heterozygous for one mutated FDD allele of *BRI2/ITM2b*. FDD_KI_ mice develop progressive synaptic and memory deficits due to loss of Bri2 function, with no amyloidosis and tauopathy. BRI2 binds APP and inhibits APP processing; owing to the loss of BRI2, APP processing is increased in FDD. Remarkably, memory deficits of FDD_KI_ mice require APP, providing genetic evidence that APP and BRI2 functionally interact, and that APP mediates FDD neuropathology.

## Results

APP processing is genetically linked to AD pathogenesis, which is consistent with a common mechanism involving toxic APP metabolites in both dementias and prompts the question whether blocking APP processing ameliorates FDD. Here we show that either a BRI2-derived peptide that, like BRI2, binds APP and blocks its cleavage by β-secretase, or a β-secretase inhibitor, rescues synaptic and memory deficits of FDD_KI_ mice. β-cleavage of APP yields two metabolites, amino terminal soluble APPβ (sAPPβ) and β-carboxyl terminal fragment (β-CTF). Further processing of β-CTF by γ-secretase cleavage releases amyloid-β, which is reputed to be central to AD pathophysiology. Intriguingly however, inhibition of γ-secretase did not ameliorate the synaptic and memory deficits of FDD mice.

## Conclusions

This result demonstrates that APP proteolysis triggers FDD, and establishes the importance of β-processing of APP for dementia to occur. Moreover, the data indicated instead that sAPPβ and/or β-CTF, rather than amyloid-β, are the toxic species causing dementia. This suggests that reducing β-cleavage of APP is an appropriate therapeutic approach to treating human dementias, while discouraging targeting γ-secretase cleavage of APP and/or amyloid-β. The recurring failures of anti-amyloid-β therapies in humans are consistent with this hypothesis.

